# Computational Performance and Statistical Accuracy of *BEAST and Comparisons with Other Methods

**DOI:** 10.1093/sysbio/syv118

**Published:** 2016-01-28

**Authors:** Huw A. Ogilvie, Joseph Heled, Dong Xie, Alexei J. Drummond

**Affiliations:** ^1^Evolution, Ecology and Genetics, Research School of Biology, The Australian National University, Canberra, Australia;; ^2^Department of Computer Science, University of Auckland, Auckland, New Zealand;; ^3^Allan Wilson Centre for Molecular Ecology and Evolution, University of Auckland, Auckland, New Zealand;

**Keywords:** Bayesian phylogenetics, Concatenation, Gene tree, Multispecies coalescent, Phylogenomics, Species tree, Supermatrix

## Abstract

Under the multispecies coalescent model of molecular evolution, gene trees have independent evolutionary histories within a shared species tree. In comparison, supermatrix concatenation methods assume that gene trees share a single common genealogical history, thereby equating gene coalescence with species divergence. The multispecies coalescent is supported by previous studies which found that its predicted distributions fit empirical data, and that concatenation is not a consistent estimator of the species tree. *BEAST, a fully Bayesian implementation of the multispecies coalescent, is popular but computationally intensive, so the increasing size of phylogenetic data sets is both a computational challenge and an opportunity for better systematics. Using simulation studies, we characterize the scaling behavior of *BEAST, and enable quantitative prediction of the impact increasing the number of loci has on both computational performance and statistical accuracy. Follow-up simulations over a wide range of parameters show that the statistical performance of *BEAST relative to concatenation improves both as branch length is reduced and as the number of loci is increased. Finally, using simulations based on estimated parameters from two phylogenomic data sets, we compare the performance of a range of species tree and concatenation methods to show that using *BEAST with tens of loci can be preferable to using concatenation with thousands of loci. Our results provide insight into the practicalities of Bayesian species tree estimation, the number of loci required to obtain a given level of accuracy and the situations in which supermatrix or summary methods will be outperformed by the fully Bayesian multispecies coalescent.

## Introduction

In recent years, a number of new techniques have applied next-generation sequencing to phylogenetics and phylogeography (McCormack et al. 2013). These new methods include target enrichment strategies (Mamanova et al. 2010) like exon capture (Bi et al. 2012), anchored phylogenomics (Lemmon et al. 2012), and ultra-conserved elements (Faircloth et al. 2012), as well as RAD sequencing (Baird et al. 2008; Davey et al. 2011). As a result, genome-wide samples of large numbers of loci from multiple individuals and multiple species have become increasingly common. This trend is rapidly shifting the *modus operandi* of systematic biology from phylogenetics to phylogenomics. This move to phylogenomics has also heralded a rapid development and uptake of species tree inference methods that acknowledge and model the discordance among individual gene trees. As with the field of phylogenetics, there is a broad acceptance that probabilistic model-based methods are preferable; however, the amount of data produced by next-generation technologies has also spurred the development of faster methods that do not utilize all the available data and employ statistical shortcuts such as admitting no uncertainty in individual gene trees (Kubatko et al. 2009; Liu et al. 2009).

### Bayesian Species Tree Estimation

The theory of incomplete lineage sorting and its implications for phylogenetic inference has been appreciated for some time (Pamilo and Nei 1988), and early approaches to applying this theory inferred the species tree that minimizes deep coalescences using gene tree parsimony (Maddison 1997; Page and Charleston 1997; Slowinski and Page 1999). The fully probabilistic application of the theory to molecular sequence analysis has only begun more recently with the introduction of Bayesian implementations of the multispecies coalescent (Rannala and Yang 2003; Edwards et al. 2007; Liu 2008; Liu et al. 2008; Heled and Drummond 2010). This model embeds gene trees within a birth–death or pure Yule species tree, and within each lineage (or branch) of the species tree, gene trees are assumed to follow a coalescent process (Heled and Drummond 2010). Prior to the development of these methods, it was necessary to assume that the history of each gene is shared and equal to the history of the species tree being studied.

However, gene trees evolve within a species tree and the approximation of equating them becomes increasingly problematic as one samples more loci, when in reality each have distinct gene tree topologies and divergence times. The multispecies coalescent brings together coalescent and birth–death models of time-trees into a single model. It describes the probability distribution of one or more gene trees that are nested inside a species tree. The species tree describes the relationship between the sampled species, or sometimes, sampled populations that have been separated for long periods of time relative to their population sizes. In the latter case it may be referred to as a *population tree* instead.

The initial implementations of the multispecies coalescent made very simple assumptions including no recombination within each locus and free recombination between loci. Although these simple assumptions can be robust to violation, including some forms of gene flow (Heled et al. 2013) (but see Leaché et al. 2014), researchers have begun to acknowledge that additional processes (such as hybridization) may need to be incorporated (Joly et al. 2009; Kubatko 2009; Chung and Ané 2011; Yu et al. 2011; Camargo et al. 2012). A number of simulation studies have also looked at various facets of performance of Bayesian species tree estimation including the influence of missing data (Wiens and Morrill 2011), the influence of low rates and rate variation among loci (Lanier et al. 2014) and comparisons of performance with “supermatrix” concatenation approaches (DeGiorgio and Degnan 2010; Larget et al. 2010; Leaché and Rannala 2011; Bayzid and Warnow 2013).

Although these modeling advances are exciting, in the face of a next-generation data deluge, this study asks and answers the following, heretofore unanswered questions: (i) How do fully Bayesian multispecies coalescent methods scale to data sets of hundreds of loci? (ii) How much more accurate will phylogenetic species tree estimates be with more sequence data? (iii) When should one use a multispecies coalescent approach instead of computationally more efficient Bayesian supermatrix approaches, or summary methods which do not use all available data? To address the first of these questions, we investigate the computational performance of the *BEAST implementation of the multispecies coalescent (Heled and Drummond 2010), so as to assess the feasibility of conducting phylogenomic analyses using existing computational tools. To shed light on the second question, we investigate how estimation accuracy improves with increasing loci.

To address the final question, we investigate how the statistical accuracy of the multispecies coalescent compares with concatenation across a broad range of conditions. We also investigate the statistical accuracy of the multispecies coalescent, supermatrix and summary methods using simulations based on two published sequence data sets; RAD tag sequences from a study of the Sino-Himalayan plant clade *Cyathophora* (Eaton and Ree 2013), and RNA-seq assemblies from a study of primates (Perry et al. 2012). *Cyathophora*, a section of the genus *Pedicularis* originating in the late Miocene or the Pliocene, is probably no older than 8 Ma (Yang and Wang 2007) and is therefore a shallow study system. In contrast, primates are a deep study system, as the oldest split in this order is estimated to have occurred in the Cretaceous around 80 Ma (Tavaré et al. 2002; Steiper and Young 2006; Wilkinson et al. 2011).

## Methods

Using simulation, we investigated the trends in computational performance and statistical accuracy of the multispecies coalescent model as implemented in BEAST 2 (*BEAST), and its statistical accuracy relative to other methods of species tree inference. In designing these simulation studies there were a number of parameters to consider. The key parameters that might determine performance of inference under the multispecies coalescent are as follows:

n : The number of species.
$n_{i}$ : The number of individuals sampled per species.
$n_{l}$ : The number of independent loci.
$n_{s}$ : The number of sites in a single locus.
$N_{e}$ : The effective population sizes of extant and ancestral species.
τ : The branch lengths in units of time or expected substitutions.

Another factor which may influence *BEAST performance is whether the molecular evolution of each locus has been more or less clock-like. Of all these parameters it is the number of loci $n_{l}$, the number of sites in a single locus $n_{s}$, and the number of individuals per species $n_{i}$ that are largely determined by experimental design. In addition, a complete specification of a multispecies coalescent model requires a speciation model (parameterized model of the species tree), a substitution model (model of the relative rates and base frequencies), and a clock model describing the absolute rate of evolution across the branches of each gene tree. In the following sections we describe the choices of parameters, models, and simulation conditions for our computational experiments.

Species and gene trees for all experiments were simulated using biopy (http://www.cs.auckland.ac.nz/~yhel002/biopy/, last accessed December 25, 2015), which simulates gene trees contained within species trees according to the multispecies coalescent process. Sequence alignments were also simulated using biopy for experiments 1 and 2, and Seq-Gen (Rambaut and Grass 1997) was used to simulate nucleotide alignments for experiment 3.

### Experiment 1: Performance of *BEAST with Increasing Numbers of Loci

The first set of simulations we performed was primarily aimed at understanding the effect that increasing the number of loci has on the computational performance and statistical accuracy of Bayesian species tree estimation. We simulated 100 random (rapidly speciating) species trees of each of three different sizes, n=5,8,13, using the birth–death process (Kendall 1948; Nee et al. 1994; Gernhard 2008). In all cases, the speciation rate was λ=1 and the extinction rate was μ=0.2 (nominally per million years). For 5-species trees we considered $n_{i}=2,4,8$, for 8-species trees $n_{i}=2,4$ and for 13-species trees $n_{i}=2$. For each combination of n and $n_{i}$ we simulated up to 256 gene trees. Gene alignments were simulated from these gene trees using an HKY substitution model (Hasegawa et al. 1985) and a strict clock. All sequences were simulated with a substitution rate of 1% per lineage per million years, a transition/transversion ratio κ of 4, equal base frequencies and a strict clock. For each *BEAST analysis, the substitution rate was fixed at 1%, and a single κ value and set of base frequencies for all loci was estimated. The locus length was 200 sites each to mimic short-read next-generation sequence data. Finally, we drew successively larger subsets of each group of alignments to form a set of *BEAST analyses (Heled and Drummond 2010). We considered increasing numbers of loci on a logarithmic scale, that is $n_{l}\in \{2,4,8,16,32,64,128,256\}$.

If the effective sample size (ESS) of either the log posterior or the age of the species tree in an analysis was not ≥200 after the initial MCMC chain was completed, we used the *resume* function in BEAST 2 (Bouckaert et al. 2014) to extend the MCMC chain from the final state of the previous run, until sufficient samples were obtained to achieve a minimum ESS of 200. For each combination of $n_{l}$, n and $n_{i}$, MCMC chains were resumed until at least 90 out of 100 replicates had sufficient ESS values. All statistics and trees were logged at a sampling rate of 1 sample per 25,000 states, and the MCMC chains that needed extension were combined into a single long chain. Pseudocode for the experimental protocol can be found in Algorithm S1 in Supplementary Material on Dryad at http://dx.doi.org/10.5061/dryad.02tf9.

ESS per hour was not calculated using the total CPU time for the combined chain because resumed runs were not restricted to a single type of CPU and hence were not directly comparable. Instead, the initial MCMC chain for each condition and replicate was restricted to a single type of CPU (Intel E5-2680 @ 2.70 GHz), and million states per hour of CPU time was calculated based on the number of states and CPU time of the initial chain. To calculate ESS per million states, the ESS of the age of the species tree was divided by the million post-burnin states in the combined chain. To calculate ESS per hour, ESS per million states was multiplied by million states per hour. All replicates were used to calculate average ESS rates, including those with ESS values <200.

The main measure of error used in this study, “relative species tree error,” incorporates both topological and branch length error by building on the previously described measure “rooted branch score” (RBS; Heled and Bouckaert 2013). Given two trees $T_{1}$ and $T_{2}$, the sets of monophyletic clades c present in each tree are defined as ${\mathbb{C}}_{1}$ and ${\mathbb{C}}_{2}$. The length of the branch which extends rootward from the most recent common ancestor (MRCA) of a clade is defined as b(c). Given these definitions, the rooted branch score is defined as the sum of all absolute differences in branch lengths b(c) between trees $T_{1}$ and $T_{2}$:

(1) $$\text{RBS}(T_{1},T_{2})=\sum_{c\in {\mathbb{C}}_{1}\cup {\mathbb{C}}_{2}}|b^{\left(1\right)}(c)-b^{\left(2\right)}(c)|.$$

By convention, the branch length of a clade that is missing from a tree is zero, so the topological error of absent or erroneous clades will be weighted by the true or estimated branch length respectively. We define the relative species tree error $e_{T}$ to be the posterior expectation of the rooted branch score distance RBS between the estimated species tree $\hat{T}$ and the true species tree $T_{true}$, normalized by the tree length of the true species tree $L_{true}$:

(2) $$e_{T}=\frac{\frac{1}{k}\cdot \sum_{i=1}^{k}\text{RBS}(T_{true},{\hat{T}}_{i})}{L_{true}}.$$

This measure summarizes the error over the entire posterior distribution by averaging the RBS for each i posterior sample ${\hat{T}}_{i}$ drawn from the entire set of posterior samples of size k. We normalize by the length of the true species tree to make the error comparable between species trees of differing units and/or number of species. Replicates with insufficient ESS values were excluded when calculating average relative species tree error, because the posterior distributions of species trees for those replicates might be inadequately sampled.

A post hoc analysis was performed to investigate the residual variation in ESS rates and relative species tree error, after accounting for the number of loci, individuals and species in each replicate. Spearman's rank correlation was used to calculate correlation coefficients between the residuals and various tree and alignment parameters. P-values for each correlation were computed using asymptotic *t* approximation, and then corrected for multiple comparisons based on 48 tests per set of residuals (Benjamini and Hochberg 1995).

Mean population size was calculated as the mean of all per-branch effective population sizes. Species tree asymmetry is the variance $\sigma_{N}^{2}$ in the number of nodes between each tip and the tree root (Kirkpatrick and Slatkin 1993). Mean tree height difference is the mean difference in height between each gene tree and the species tree. Mean deep coalescences is the mean number of deep coalescences for each gene as calculated by DendroPy 4.0.3 (Sukumaran and Holder 2010). The mean parsimonious mutations is the parsimonious (minimum) number of mutations required per site given the true gene tree, again calculated by DendroPy. Mean variable site count is the mean number of sites per locus with more than one extant allele, and mutations per variable site is the total number of parsimonious mutations required divided by the total number of variable sites.

Experiment 1 was performed using the Pan cluster provided by New Zealand eScience Infrastructure and hosted at the University of Auckland (http://www.eresearch.auckland.ac.nz/en/centre-for-eresearch/research-facilities/computing-resources.html, last accessed December 25, 2015). This high performance compute cluster provides access to Linux compute nodes with 2.7 and 2.8GHz Intel Xeon CPUs, and approximately 8 GB of RAM per CPU core.

### Experiment 2: Comparing a Bayesian Multispecies Coalescent Approach with a Bayesian Supermatrix Approach

In the second set of simulations, we compare the statistical accuracy of the multispecies coalescent to partitioned concatenation, both as implemented in BEAST 2. We refer to these methods as *BEAST and Bayesian supermatrix respectively. Specifically we tested the hypothesis that the comparative accuracy would depend on mean branch length in coalescent units of $\tau {\left(2N_{e}\right)}^{-1}$.

For every combination of n=4,5,6,8 and $n_{l}=1,2,4$, we simulated species trees with a range of branch lengths in coalescent units. In order to vary branch lengths, species trees were simulated with expected root heights of $R=\frac{1}{2},1,2,4,8,16$ (nominally in millions of years) and population sizes chosen from $N_{e}=\frac{1}{4},\frac{1}{2},1$ (nominally in units of million individuals), changing the coalescent branch length unit numerator and denominator respectively. Additional expected root heights were included where the most accurate method switches from *BEAST to Bayesian supermatrix, to obtain denser sampling in that part of parameter space.

Species trees were generated under the pure birth Yule model (Yule 1924). The birth rate for each combination of parameters was set to $\lambda =\frac{1}{R}\sum_{k=2}^{n}\frac{1}{k}$, that is, the birth rate which generates trees with an expected root height of R. These settings roughly correspond to mammalian nuclear genes of species with an effective population size of one-quarter, one half or one million individuals.

A single individual per species was simulated for all loci. We used the Jukes–Cantor substitution model (Jukes and Cantor 1969) and a strict clock model for each locus, but with rate variation between loci. The mutation rate for the first locus was fixed at $\mu_{0}=0.01$, and the rates for other loci drawn from the range $\left[\mu_{0}/F,\mu_{0}\times F\right]$. We used F=3, giving a factor of 9 between the fastest and slowest possible rates. The rate was drawn in log space, so there is equal density of slower and faster rates around $\mu_{0}$. The number of sites per alignment ($n_{s}$) was fixed at 1000.

We generated 100 replicates for each combination of n, $n_{l}$, R and $N_{e}$. For each unique combination of n, R and $N_{e}$ only one set of 100 species trees was generated and used (regardless of $n_{l}$) to minimize species tree sampling error when analyzing the effect of increasing $n_{l}$. Gene trees and extant sequences were generated separately for each replicate and for each value of $n_{l}$.

Both Bayesian supermatrix and *BEAST analyses used a Yule prior on the species tree, with a uniform prior of ${[}^{1}{/}_{100},100]$ on λ, and a separate partition per locus each with a strict clock model, where the clock rate of the first partition was fixed to the truth ($\mu_{0}$) and the other rates were estimated. The *BEAST effective population size hyperparameter (popMean) was given a uniform prior in the range $\left[\frac{1}{5},5\right]$, and all population sizes were estimated.

The Bayesian supermatrix analysis used a fixed chain length of 4 million states, sampling every 1000 states. The *BEAST analysis used a fixed chain length of 40 million states, sampling every 10,000 states. The ESS values of the posterior, likelihood and prior statistics of each chain were estimated, and replicates where the ESS was <200 for any of those statistics were discarded. For each combination of n, $n_{l}$ and method there were never more than 4% of replicates discarded for this reason (Figure S10 in Supplementary Material available on Dryad). As with experiment 1, this experiment was performed using the NeSI Pan cluster.

### Experiment 3: Many-method Comparison of Species Tree Inference using Parameters Estimated from Two Phylogenomic Data Sets

The purpose of the third set of simulations was two-fold: to check that the trends in statistical accuracy observed for the first two sets of simulations held for empirically derived simulations, and to compare statistical accuracy across a range of species tree inference methods. To simulate more realistic trees and sequences, we derived a range of properties and phylogenetic parameters from two empirical phylogenomic data sets for use as simulation parameters.

The biallelic species tree inference method SNAPP (Bryant et al. 2012) was used to estimate speciation birth rates and effective population sizes because it did not require phasing the sequence data. To estimate base frequencies, substitution rates, between-site rate variation, and between-locus rate variation, we used a Bayesian supermatrix analysis with a Yule prior on the species tree. A detailed description of sequence data processing and SNAPP and BEAST settings is given in Supplementary Material available on Dryad.

We simulated 100 replicates each of “deep” and “shallow” Yule species trees of n=12 and n=8 respectively, using the inferred empirical birth rates, with per-branch population sizes picked from a gamma distribution of shape 2 and a mean equal to the mean inferred population sizes. For the deep species trees we simulated 512 gene trees, and for the shallow species trees we simulated 4096 gene trees within each species tree, each with two individuals per species.

For each simulated gene tree, we chose a strict clock rate from the gamma distribution defined by the inferred shape parameters and scale parameters. Nucleotide sequences were simulated for every locus using the empirically derived GTR+G base frequencies, substitution rates, and gamma rate variation from the applicable study. As the shallow study used 64nt RAD tags, we picked that fixed length for sequence simulations based on that study. For simulations based on the deep study, each simulated alignment length was randomly sampled (with replacement) from the original alignment lengths of the deep study.

Species trees were reconstructed from simulated sequences using five different multi-locus inference methods; *BEAST, Bayesian supermatrix, MP-EST (Liu et al. 2010), RAxML version 8 (Stamatakis 2014), and BIONJ (Gascuel 1997). We tested *BEAST performance given $n_{l}=1,2,4,8$ for the deep study based simulations and $n_{l}=1,2,4,8,16,32$ for the shallow study based simulations. For all simulations, we tested the performance of Bayesian supermatrix given $n_{l}=1,2,4,8,16,32,64,128,256,512$. For the deep study simulations we tested RAxML, BIONJ, and MP-EST with $n_{l}=1,2,4,8,16,32,64,128,512$. For the shallow study simulations, we also analyzed $n_{l}=1024,2048,4096$. Both *BEAST and MP-EST can infer species trees utilizing more than one individual per species, and we tested both methods using $n_{i}=1,2$.

All GTR + G rates were estimated for *BEAST and Bayesian supermatrix analyses. For RAxML analyses, only GTR + G substitution rates were estimated and empirical base frequencies were used. Clock rate distribution parameters and clock rates for each locus were estimated for *BEAST and Bayesian supermatrix analyses. Loci were not partitioned for RAxML analyses, so per-locus clock rates could not be estimated for that method. The RAxML maximum likelihood algorithm used was “new rapid hillclimbing.” Pairwise distances matrices calculated by RAxML were used to generate neighbor-joining trees using the BIONJ algorithm implemented in PAUP* version 4.0a142 (http://paup.csit.fsu.edu/, last accessed December 25, 2015). *BEAST and BEAST trees are implicitly rooted because they are ultrametric, and RAxML and BIONJ trees were midpoint rooted.

MP-EST uses gene trees as input data, which were inferred using RAxML. The same settings used for RAxML species tree inference were used for gene tree inference, and gene trees were midpoint rooted. For each replicate MP-EST was set to make 10 independent runs, and the species tree with the highest pseudo-likelihood was retained for further analysis.

The BEAST and *BEAST chains were run on the Raijin cluster provided by the National Computational Infrastructure (http://nci.org.au/systems-services/national-facility/peak-system/raijin/, last accessed December 25, 2015). This cluster provides access to Linux compute nodes with 2.6 GHz Intel Xeon Sandy Bridge CPUs, and 4 GB of RAM was requested per run. Further details of BEAST and *BEAST chains are provided in Supplementary Material available on Dryad. RAxML and MP-EST were run on the cluster provided by the Genome Discovery Unit of the Australian Cancer Research Foundation Biomolecular Resource Facility. Jobs on this cluster ran on Linux compute nodes with a variety of Intel Xeon and AMD Opteron CPUs, and 2 GB of RAM was requested per RAxML or MP-EST job.

## Results

### Experiment 1: Performance of *BEAST with Increasing Numbers of Loci

#### Computational performance

**Figure 1.**
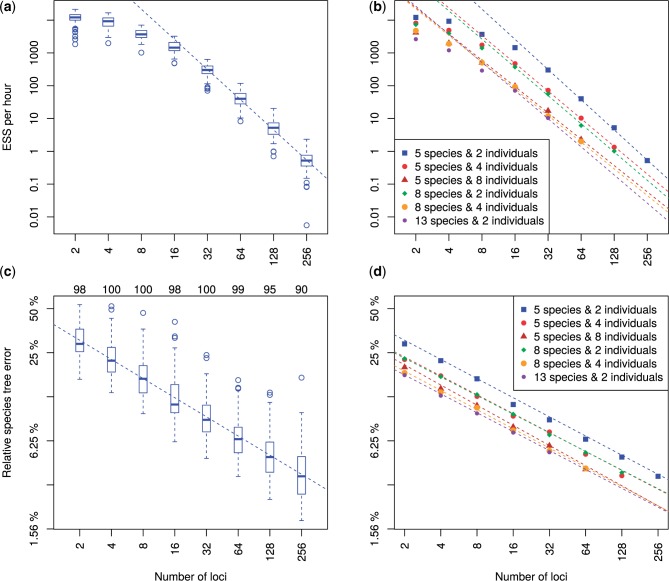
Trends in ESS per hour and relative species tree error as a function of the number of loci. a) ESS per hour for analyses of 5 species each with 2 individuals. Each box-and-whisker shows the variance in mixing across a hundred replicate data sets for each number of loci. b) The median ESS per hour as a function of number of loci, with trend lines for each combination of number of species and individuals per species. Solid shapes indicate the median value for each category, and regression lines were calculated using all replicates for each category. c) Relative error for 5 species each with 2 individuals, with each box-and-whisker showing the variance in relative error between replicates. Numbers above the graph area indicate how many replicates were included for each number of loci. d) The relative error in the estimated species tree as a function of the number of loci, with trend lines for each combination of number of species and individuals per species. Solid shapes indicate the median value for each category, and regression lines were calculated using all replicates for each category with sufficient ESS.

We evaluated the scaling of computational performance of *BEAST as a function of the number of loci analyzed. We recorded the elapsed computational time for each replicate analysis running in a single thread. This was then used to calculate the effective number of samples per hour (ESS per hour), to measure the computational effort required to produce a sample from the posterior for a given number of loci. The ESS per hour relationship (Fig. 1a, S3 in Supplementary Material available on Dryad) suggests that a power law fits the scaling of computational performance. The linear relationship in the log-log plot indicates that a power law fits well for the range from 32 to 256 loci. We extrapolate that for n=5, $n_{i}=2$ and $n_{l}\geq 32$, ESS per hour follows a power law with a slope and intercept of −3.06±0.04 and 16.34±0.18, respectively.

Applying this functional relationship, we could estimate the computational cost to analyze a similar data set with a larger number of loci. For example, given 5 species and 2 individuals in the simulation, the predicted ESS per hour is 0.54 for 256 genes, which indicates it would take approximately 369 CPU hours to attain an ESS of 200. We can therefore estimate that a similar analysis of 1024 loci would take roughly 1064 CPU days. Nevertheless, an analysis of this size might be achieved within 2 months by parallelizing the problem into 20 independent MCMC chains for 2 months each and discarding a few days of burnin from each of them, to achieve on the order of 10 independent samples from each chain.

Variation in ESS per hour between replicates was observed under all tested conditions (Figure S3 in Supplementary Material available on Dryad). The slowest replicate relative to the median rate for any condition was a 5 species, 2 individuals and 256 genes outlier, 94× slower than the median rate for that combination (Fig. 1a). This replicate would require approximately 1500 CPU days to attain an ESS of 200. However, this was an extreme case as the next slowest replicate for that combination was another outlier only 6.4× slower than the median rate, and would require only 100 CPU days to attain the same ESS value.

The slope of the expected computational performance as a function of number of loci does not vary with the number of species or the number of individuals (Fig. 1b), although a larger range of n and $n_{i}$ would need to be examined to understand the scaling relationship of computational performance with those quantities. For analyses larger than 5 species and 2 individuals, the power law range appears to begin at $n_{l}\geq 16$. Combining all simulation results, a multiple linear regression describing a response variable Y (e.g., ESS per hour) as a function of three explanatory variables: number of loci $n_{l}$, number of species n, and number of individuals per species $n_{i}$, can be constructed as follows:

(3) $$\log(Y)=\beta_{1}log(n_{l})+\beta_{2}n+\beta_{3}n_{i}+\alpha .$$

Taking the ESS per hour as the response variable, the linear regression estimates of the coefficients are $\beta_{1}=-2.81\pm 0.02$, $\beta_{2}=-0.42\pm 0.01$, $\beta_{3}=-0.46\pm 0.01$, and the intercept is α=17.98±0.13. At least within the range of parameters examined here, it appears that the $\beta_{1}$ coefficient is not greatly influenced by n and $n_{i}$ (Fig. 1b).

We also considered the scaling of the number of effective samples per million states (ESS per million states) in the MCMC analyses. This quantity is complementary to our first result; it is easier to investigate as it does not require running all simulations on identical and dedicated hardware. Computational time for methods like *BEAST is dominated by the phylogenetic likelihood, which is calculated for all site patterns given a proposed tree (Yang et al. 1994). Because *BEAST infers a separate gene tree for each locus, the time per state will be linear with the number of loci assuming the average number of site patterns per locus is independent of the total number of loci. This assumption of independence holds for experiment 1 because loci were subsetted uniformly.

Adapting the terminology of Equation (3), the slope of ESS per hour ($\beta_{1h}$) will be simply related to the slope of ESS per million states ($\beta_{1s}$): $\beta_{1h}=\beta_{1s}+1$. However because CPU time per site pattern depends on the specific hardware employed, the intercept of ESS per hour ($\alpha_{h}$) cannot be predicted from that of ESS per million states ($\alpha_{s}$).

As expected, ESS per million states also exhibits a power law in the number of loci (Figure S4 in Supplementary Material available on Dryad). By assigning the ESS per million states to Y in the multiple linear regression in Equation 3, the estimated coefficients are $\beta_{1}=-1.87\pm 0.02$, $\beta_{2}=-0.28\pm 0.01$, $\beta_{3}=-0.24\pm 0.01$, and the estimated intercept is α=9.07±0.12. The difference in slope between ESS per million states and ESS per hour is (−1.87)−(−2.81)=0.94, very close to 1 as predicted. As with ESS per hour, observations used for the linear regression were restricted to $n_{l}\geq 32$ for the 5 species, 2 individual case and $n_{l}\geq 16$ for other cases.

Using the example of 5 species and 2 individuals, the slope and intercept are −1.97±0.04 and 7.86±0.18 respectively, so the predicted ESS per million states for 256 individuals is 0.047 (Figure S4a in Supplementary Material available on Dryad). It would therefore take approximately 4.3 billion states to obtain an ESS of 200. We can extrapolate that a similar analysis of 1024 loci would require an MCMC chain of roughly $4.3\times {\left(\frac{1024}{256}\right)}^{1.97}\approx 66$ billion states.

#### Statistical accuracy

We also calculated the relative error in the species tree estimate for each replicate. For some larger analyses it was challenging to achieve acceptable ESS values for every replicate, even with chain lengths of several billion states and access to high-performance computational infrastructure. To retain the larger analyses without biasing statistical accuracy, we excluded replicates in which the ESS of either the log posterior or the species tree age was smaller than 200. All remaining replicates were used for a linear regression analysis of the contribution of the number of loci to relative species tree error. This analysis revealed a power law relationship from 2 to 256 loci (Fig. 1c, S5 in Supplementary Material available on Dryad). Given 5 species and 2 individuals, the slope and intercept are −0.435±0.007 and −0.889±0.026 respectively, so the relative species tree error predicted by the power law for 256 loci is 0.037. By extrapolation, we would therefore estimate that the relative error of a 1024 loci analysis would decrease to $0.037\times {\left(\frac{1024}{256}\right)}^{-0.435}\approx 0.020$.

Linear regression analysis of relative species tree error for all combinations of n and $n_{l}$ showed little variation in the trend line slope between conditions (Fig. 1d). By assigning the relative species tree error to Y in the multiple linear regression in Equation (3), the estimated coefficients are $\beta_{1}=-0.433\pm 0.003$, $\beta_{2}=-0.066\pm 0.002$, $\beta_{3}=-0.070\pm 0.002$, and the estimated intercept is α=−0.481±0.022. More details for all multiple linear regression models are available in Supplementary Material available on Dryad. Trends in topology-only accuracy inferred using rooted Robinson-Foulds (rRF) scores are also presented in Supplementary Material as Dryad, Figure S9 and Table S12 available on Dryad.

Finally, we also analyzed the number of species tree topologies sampled in each posterior distribution. It appears that for the analyses involving 8 and 13 species there is a rapid reduction in the number of topologies in the 95% credible set with increasing numbers of loci, but it does not follow a power law (Figure S7 in Supplementary Material available on Dryad).

#### Post hoc analysis of convergence and species tree error

Experiment 1 was designed to investigate the relationship between the number of loci $n_{l}$, number of species n and number of individuals $n_{i}$ on ESS rates and statistical accuracy. Although these variables explained most of the variation in ESS rates and accuracy, residual variation was present between the 100 replicates of each combination of $n_{l}$, n and $n_{i}$ (Fig. 1a and c). The correlations between this residual variation and a collection of phylogenetic statistics that could be extracted from the simulated trees and alignments were studied in a post hoc analysis.

The only tree or alignment statistic that was significantly correlated with ESS per hour consistently across all conditions was mean tree height difference (Table 1). This statistic is the mean difference in height between each gene tree and the species tree. The positive correlation observed for this parameter suggests that when gene trees are taller relative to the species tree, the ESS rate will be higher and *BEAST will converge more quickly.

**Table 1.** Spearman correlation of tree and alignment parameters with ESS per hour

	5n, $2n_{i}$	5n, $4n_{i}$	5n, $8n_{i}$	8n, $2n_{i}$	8n, $4n_{i}$	13n, $2n_{i}$
Species tree height	0.068	0.222***	0.362***	−0.036	0.180***	0.120
Mean population size	0.075	−0.048	−0.086	−0.020	−0.101	0.121
Species tree asymmetry	−0.238***	−0.088	−0.045	−0.125*	0.013	−0.068
Mean deep coalescences	−0.122**	−0.225***	−0.295***	0.020	−0.079	0.044
Mean parsimonious mutations	0.099	0.148***	0.122*	−0.013	0.124*	0.074
Mean variable site count	0.088	0.228***	0.294***	−0.045	0.146**	0.042
Mean tree height difference	0.246***	0.355***	0.315***	0.421***	0.340***	0.398***
Mutations per variable site	0.030	−0.066	−0.123*	0.046	0.016	0.057

*P<0.05, **P<0.01, ***P<0.001.

In contrast to ESS per hour, several statistics were consistently significantly correlated with relative species tree error (Table 2). The height of the species tree and the number of variable sites per locus were negatively correlated with relative error. This result is somewhat intuitive, as taller species trees will have longer branches which are easier to resolve, and the number of variable sites is an obvious proxy for the amount of information in each locus. Relative error was positively correlated with the mean number of deep coalescences and the number of mutations per variable site. Those correlations suggest that data sets with more incomplete lineage sorting will be more difficult to resolve, and that saturated sites may increase uncertainty.

**Table 2.** Spearman correlation of tree and alignment parameters with species tree error

	5n, $2n_{i}$	5n, $4n_{i}$	5n, $8n_{i}$	8n, $2n_{i}$	8n, $4n_{i}$	13n, $2n_{i}$
Species tree height	−0.734***	−0.582***	−0.330***	−0.702***	−0.537***	−0.580***
Mean population size	0.103*	0.078	0.006	0.118*	0.004	0.076
Species tree asymmetry	0.041	0.011	0.035	−0.170***	−0.181***	−0.050
Mean deep coalescences	0.665***	0.573***	0.273***	0.647***	0.522***	0.591***
Mean parsimonious mutations	−0.387***	−0.199***	−0.025	−0.372***	−0.184***	−0.378***
Mean variable site count	−0.587***	−0.494***	−0.242***	−0.607***	−0.530***	−0.642***
Mean tree height difference	0.194***	0.186***	0.196***	0.173***	0.207***	0.127*
Mutations per variable site	0.416***	0.306***	0.152**	0.333***	0.220***	0.148*

*P<0.05, **P<0.01, ***P<0.001.

### Experiment 2: Statistical Accuracy of *BEAST Relative to Bayesian supermatrix

To assess the statistical accuracy of the *BEAST relative to the standard Bayesian supermatrix approach, we conducted a simulation study where we simulated species trees with a broad range of mean branch lengths for varying numbers of species and loci. Gene coalescences occur prior to species divergence times, and the severity of this discrepancy will depend on species tree branch lengths in units of coalescent time. Because the multispecies coalescent accounts for this phenomenon but the Bayesian supermatrix approach does not, we expected the multispecies coalescent to outperform the Bayesian supermatrix approach for trees with shorter branch lengths.

The “species tree error ratio” $e_{T_{a}}/e_{T_{b}}$ is a measure of the comparative accuracy and is specified as follows, where a is *BEAST and b is Bayesian supermatrix:

(4) $$\frac{e_{T_{a}}}{e_{T_{b}}}=\frac{\frac{1}{k_{a}}\cdot \sum_{i=1}^{k_{a}}RBS(T_{true},{\hat{T}}_{ai})}{\frac{1}{k_{b}}\cdot \sum_{i=1}^{k_{b}}RBS(T_{true},{\hat{T}}_{bi})}.$$

**Figure 2.**
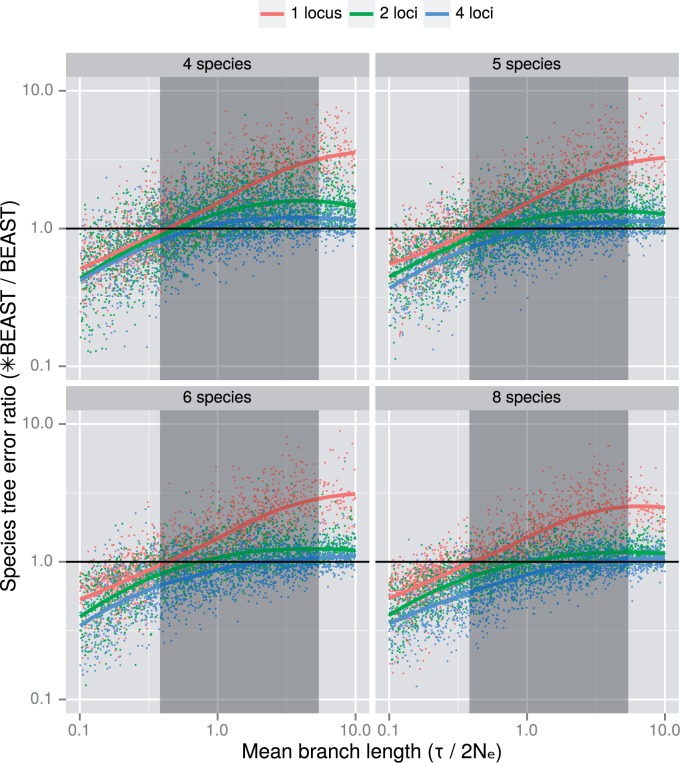
Species tree error ratio (*BEAST/BEAST) as a function of the average species tree branch length (in coalescent units) for trees of 4, 5, 6, and 8 species. Data points are below 1 (black line) where the *BEAST error is lower than the BEAST error, indicating that *BEAST was more accurate than BEAST. Data points above 1 show the opposite. Only results with both mean branch lengths and error ratios between 0.1 and 10.0 are included. The red, green, and blue lines show the local regression for one, two and four locus estimates, respectively. The shaded region indicates where the crossover point depended on the combination of simulation parameters chosen—*BEAST was always preferred for average branch lengths shorter than this zone.

Values below 1 indicate lower error, or equivalently superior accuracy, when using *BEAST instead of Bayesian supermatrix. For all numbers of species tested, the statistical accuracy of *BEAST was superior to Bayesian supermatrix for trees with shorter mean branch lengths (Fig. 2). Using LOESS regression, it is clear that as the number of loci increases, *BEAST performance improves relative to Bayesian supermatrix because for a given mean branch length, the species tree error ratio decreases as the number of loci increases (Fig. 2).

For all numbers of species and loci tested, there is a mean branch length crossover point where for shorter mean branch lengths, *BEAST is expected to outperform Bayesian supermatrix, and *vice versa* for longer mean branch lengths. The crossover point depends on the number of loci; as the number of loci increases, the point shifts right (Fig. 2), indicating that *BEAST is expected to outperform Bayesian supermatrix for a larger range of mean branch lengths, consistent with the general trend of improved performance of *BEAST when increasing the number of loci.

Within the parameter region explored in this experiment, depending on the number of species, loci and the effective population sizes, the crossover point was found in the range $0.382\tau {\left(2N_{e}\right)}^{-1}$ to $5.416\tau {\left(2N_{e}\right)}^{-1}$ (Figure S11 in Supplementary Material available on Dryad). For mean branch lengths shorter than $0.382\tau {\left(2N_{e}\right)}^{-1}$, *BEAST was preferred regardless of the parameters explored, even when using a single locus (Fig. 2). The crossover point given a single locus was always below $0.5\tau {\left(2N_{e}\right)}^{-1}$ (Figure S11 in Supplementary Material available on Dryad) and given longer mean branch lengths the relative performance of Bayesian supermatrix was higher than for multi-locus inference (Fig. 2). This implies that *BEAST is still useful for single-locus studies of species trees with short branches, but should be applied with caution.

### Experiment 3: Inferred Parameters of Phylogenomic Data Sets and Multi-method Comparison

Sequence data sets from two published studies were realigned and reanalyzed to calculate their empirical properties and phylogenetic parameters. Besides the expected difference in speciation rate (which for the shallow study rate was over six times faster, corresponding to much shorter branch lengths), the shallow plant study sequences were very AT rich, whereas the deep primate study sequences were moderately GC rich (Table 3). C⇆T substitutions were a greater proportion of all substitutions for the deep study, but the between-site gamma rate variation was flatter. The mean effective population size $N_{e}$ of the deep study was estimated to be only 2.4% that of the shallow study.

**Table 3.** Experiment 3 data set properties and mean values of inferred parameters

Phylogenetic depth	Shallow	Deep
Clade name	Cyathophora	Primates
Taxonomic rank	Section	Order
Sequence data	RAD tag	RNA-seq
In-group nS	8	12
Base frequency: A	0.290	0.266
Base frequency: C	0.212	0.240
Base frequency: G	0.204	0.263
Base frequency: T	0.294	0.231
A⇆C rate	0.367	0.152
A⇆G rate	0.940	0.694
A⇆T rate	0.246	0.100
C⇆G rate	0.305	0.155
C⇆T rate	1.000	1.000
G⇆T rate	0.353	0.127
Gamma rate variation	0.0383	0.233
Speciation birth rate	125.3	20.7
Per-branch $N_{e}$	$6.35\times 10^{-3}$	$1.53\times 10^{-4}$
Locus length	64nt	110–3511nt
Clock variation shape	6.22	5.15
Clock variation scale	0.173	0.195

All inferred parameters are rounded to three significant figures or one decimal place, whichever is more precise.

The original publication of *Cyathophora* sequences and phylogeny suggested that *P. rex* subsp. *rockii* is sister to subsp. *rex* and subsp. *lipskyana* (Eaton and Ree 2013). The most common species tree topology seen in both SNAPP and Bayesian supermatrix posterior distributions supports this placement (Figures S16 and S17 in Supplementary Material available on Dryad). The original study left open the question of *P. thamnophila* monophyly but raised the possibility that the apparent paraphyly of this species, as replicated by our reanalysis, is an artifact of introgression (Eaton and Ree 2013). Species trees inferred by SNAPP and Bayesian supermatrix from reanalysis of the deep phylogenetic study (Figure S18,S19) agreed with the accepted primate phylogeny (Perry et al. 2012).

#### Analysis of empirical-based simulations

We simulated species trees, gene trees, and sequences based on the estimated parameters of both data sets (Table 3), and refer to these simulations as shallow and deep phylogenetic simulations respectively. The mean branch length of the simulated shallow species trees was $0.539\tau {\left(2N_{e}\right)}^{-1}$, compared with $159.8\tau {\left(2N_{e}\right)}^{-1}$ for the simulated deep species trees. We computed the relative species tree error for all *BEAST analyses of these simulations.

**Figure 3.**
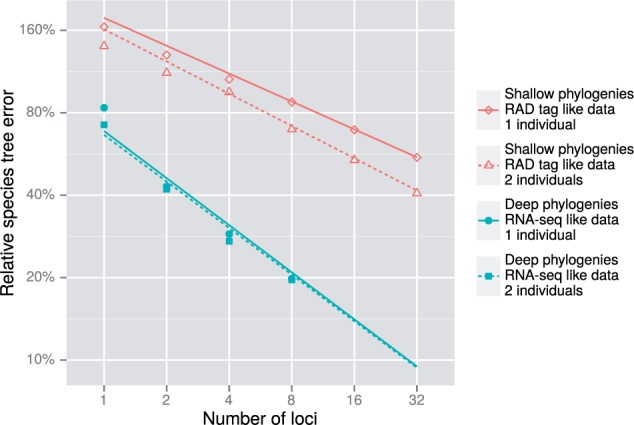
The relative species tree error as a function of the number of loci for empirical-based simulations. Both shallow and deep phylogenetic simulation results are presented. Solid and hollow shapes are the median value for each category, and regression lines were calculated using all replicates for each category.

The relative species tree errors for all values of $n_{l}$ and $n_{i}$ considered were computed for both simulation types. A power law appeared to fit the relationship between relative error and number of loci for values of $n_{l}\geq 2$, so log-log linear regression analyses were restricted to $n_{l}\geq 2$. The log-log slope connecting relative error and the number of loci appears mostly independent of $n_{i}$ for shallow phylogenetic simulations. For deep simulations, the trend lines for $n_{i}=1$ and $n_{i}=2$ were very close, implying that multiple individuals did not improve accuracy for those simulations (Figure 3).

This result is consistent with the initial set of simulations reported in “Statistical accuracy.” However, the log-log slopes varied substantially between *BEAST inference of shallow and deep phylogenetic simulations. The difference in power law exponents inferred using multiple linear regression (Tables S13 and S14 in Supplementary Material available on Dryad) between shallow and deep simulations was (−0.365)−(−0.568)=0.203.

Results from the initial simulation study, detailed in “Computational performance,” suggest that a power law relationship of ESS and number of loci only applies to *BEAST analyses of 16 to 32 loci and above. As we only inferred deep phylogenetic trees utilizing up to 8 loci and shallow phylogenetic trees up to 32 loci using *BEAST, we cannot make firm conclusions regarding the scaling laws of ESS performance using this set of simulations.

#### Alternative methods for multi-locus phylogenetic inference

The second analysis we conducted based on the empirically derived shallow and deep phylogenetic simulations was a comparison of common multi-locus methods of species tree inference. This encompassed the Bayesian multispecies coalescent (*BEAST), Bayesian supermatrix (BEAST), Maximum-likelihood supermatrix (RAxML), neighbor-joining (BIONJ), and summary coalescent (MP-EST) methods. As some methods provide only a single best tree estimate in place of a posterior distribution of trees, we used common ancestor summary trees (CAT; Heled and Bouckaert 2013) for *BEAST and Bayesian supermatrix analyses in this comparison.

**Figure 4.**
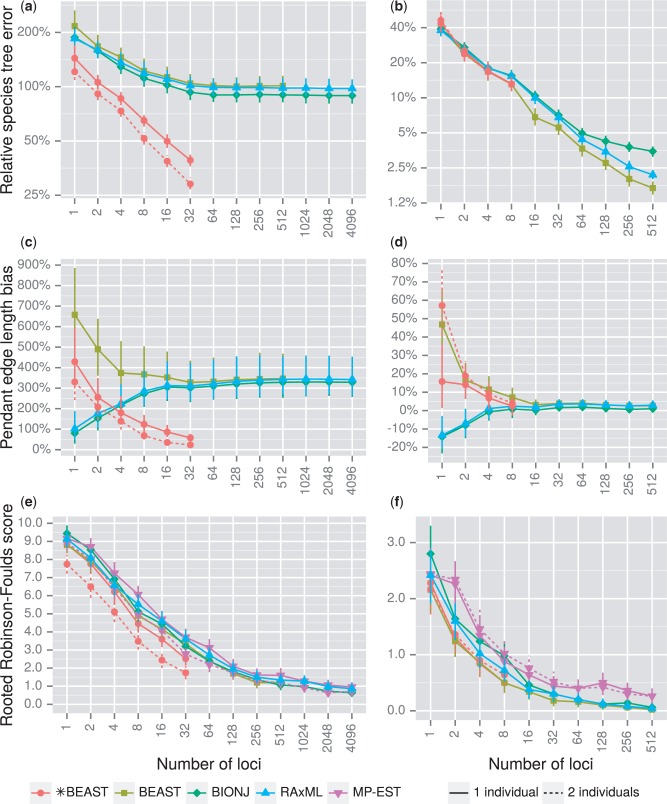
Statistical accuracy of multiple species tree inference methods as a function of the number of loci. Shallow phylogenetic simulation results (a, c, e) and deep results (b, d, f) are both presented. Measures of statistical accuracy used here are relative species tree error a) and b) which incorporates branch length and topological error, pendant edge length bias c) and d) which highlights biased branch lengths inferred by noncoalescent methods at the tips of the tree, and rooted Robinson–Foulds scores e) and f) which are a purely topological measure. All solid shapes in subfigures a–d show trimmed means (25% trim to reduce the influence of outliers), or untrimmed means for subfigures e) and f). Vertical range lines show 95% confidence intervals for each mean, calculated by bootstrapping.

Based on relative species tree error, *BEAST outperformed all other methods for any given number of loci for the shallow simulations. The statistical accuracy of Bayesian supermatrix, RAxML and BIONJ all plateaued beyond 64 loci for the shallow simulations, whereas *BEAST appears to follow a power law as previously suggested (Fig. 4a). The statistical accuracy of all methods improves with increasing numbers of loci for the deep simulations, however we limited the simulations to a maximum of 8 loci when running *BEAST. The statistical accuracy of all methods tested was similar up to 8 loci, but for larger numbers of loci Bayesian supermatrix analysis was superior and BIONJ was inferior to RAxML (Fig. 4b).

A major factor causing the poor performance of methods other than *BEAST for the shallow simulations is a bias when estimating pendant edge (also known as leaf or tip) length. Although the mean bias of estimated pendant edge length trends towards zero for *BEAST, other methods converge on a bias of approximately 350%, meaning estimated pendant edges are on average 4.5× the true length (Fig. 4c). In contrast, there is only a small positive bias using methods other than *BEAST for the deep simulations (Fig. 4d).

Relative species tree error incorporates both topological error and branch length error. To separate these two components, we calculated the mean rRF score as a measure of purely topological error—estimated topologies more distant from the truth will have higher rRF scores. For shallow simulations, *BEAST was the best-performing method, and the topological accuracy of both *BEAST and MP-EST was improved given two individuals per species (Fig. 4e). For deep simulations, all methods other than *BEAST and MP-EST converged at near-zero topological error given 512 loci (Fig. 4f). *BEAST was limited to a maximum of 8 loci, but its performance for a given number of loci was very close to Bayesian supermatrix. The topological accuracy of MP-EST was inferior to all other methods analyzed.

## Discussion and Conclusions

We have demonstrated by simulation that the multispecies coalescent (as implemented in *BEAST) can be applied to some problems involving hundreds of loci. In order to analyze the performance of *BEAST with hundreds of loci under various conditions, with 100 replicates per condition and given finite computational resources, we made choices partly based on computational expediency. These included relatively limited numbers of species and individuals, and assuming a strict molecular clock. More complexity in the sense of more parameters to estimate, for example denser taxon sampling or relaxed clocks, would be expected to require more computational time than the analyses reported here.

Researchers studying the evolutionary histories of organisms are not burdened by the need to test hundreds of replicates across many conditions, and can therefore conduct larger analyses using *BEAST. For example, a recent study of Neotropical cotingas (Cotingidae: Aves) applied *BEAST to resolve a species tree of 67 extant bird lineages, and used a lognormal relaxed clock for each locus with molecular rate calibrations to infer absolute divergence times. ESS rates for all logged statistics were greater than 200 and convergence was also confirmed graphically, demonstrating that *BEAST can be applied to real phylogenetic data sets with many taxa, and may also be used with a relaxed clock (Berv and Prum 2014).

### Power Laws Describe *BEAST Scaling Behavior

For the various numbers of species, individuals and loci analyzed in this study, power laws could be used to describe the observed trends in computational performance of *BEAST, and in the statistical accuracy of the fully Bayesian multispecies coalescent. In terms of computational performance, this provides a benchmark for the efficiency of Bayesian MCMC approaches to inference under the multispecies coalescent. Our results are a product of the particular algorithm design decisions that the authors of *BEAST have made, and we hope that power law exponents can be improved upon by subsequent efforts to produce more efficient algorithms for inference under the multispecies coalescent model.

In contrast, the power law that describes the decrease in estimation uncertainty associated with inference of the species tree with increasing number of loci is a fundamental property of the model itself, and will hold regardless of the details of the algorithmic approach to inference under this model. It therefore represents a fundamental feature of the problem of species tree inference. With these results, it is possible to extrapolate what one might expect to achieve by expanding data from a small pilot study to a more comprehensive sample of the genomic material of a set of study species or individuals.

The decrease in relative species tree error given different numbers of species and individuals was investigated in experiment 1. Other phylogenetic parameters were fixed, including the locus length, substitution model and population size distributions. Possibly because of this, the variation in power law exponents was minimal. Experiment 3 in contrast compared shallow and deep phylogenies with larger and smaller population sizes respectively, and associated alignments of short fixed-length loci and longer variable-length loci respectively. Clock rate variation and substitution model rates also differed between conditions. Power law exponents did vary between experiment 1 and both the shallow and deep inferences in experiment 3; exponents were −0.433, −0.365 and −0.568 respectively. This is important because larger exponents imply a greater decrease in relative species tree error, so additional loci will lead to a larger improvement in accuracy of inferred species trees than with a smaller exponent.

Given a hypothetical pilot study of 16 loci, it may be of interest what the decrease in error would be for a full study of 256 loci. Because the number of loci in this scenario is increased 16 times, the reduction in relative species tree error of the full study compared with the pilot study would be $1.0-16^{-0.433}\approx 70\%$ if the study is similar to experiment 1, $1.0-16^{-0.365}\approx 64\%$ if it is similar to the shallow phylogenetic simulations, or $1.0-16^{-0.568}\approx 79\%$ if it similar to the deep phylogenetic simulations. What these calculations should remind us about the power law relationship is that expanding data from 1 to 16 loci provides as great an increase in statistical accuracy as expanding from 16 to 256 loci. That is, for each subsequent locus added there is a diminishing return with regards to statistical accuracy.

The power laws describing computational performance can also be used to predict the increase in computational time and chain length required to achieve sufficient sampling of the posterior distribution. In experiment 1, the power law coefficient for the log number of loci was −2.81 for ESS per hour and −1.87 for ESS per million states. Given the previous example going from 16 to 256 loci, the amount of time required for sufficient sampling of data sets similar to experiment 1 would increase by $16^{2.81}\approx 2408$ times. The chain length (number of states) required would increase by $16^{1.87}\approx 180$ times.

Some residual variation in ESS rates was observed after accounting for the number of individuals, species, and loci in each analysis. This was unsurprising as the operators used by *BEAST are stochastic (Höhna and Drummond 2012), so even when applied to the same data ESS rates are expected to vary between runs. Consistent with this expectation, the only nonstochastic contribution identified in our post hoc analysis was a moderate correlation between residual ESS per hour and the average gene and species tree height difference.

It is possible that the parameters which were kept constant in our analysis (e.g., the substitution rate, or the number of sites per loci, or the choice of a strict molecular clock) may change the relationship between the number of loci and computational performance or statistical accuracy. Given a sequence data set with substantially different properties from experiment 1, increasing the number of loci might have a smaller or larger effect on computational performance.

### *BEAST Compared with Other Methods

A previous simulation study which analyzed the scaling behavior of *BEAST and other methods used just two species trees to report on topological accuracy given a range (5, 10, 25, and 50) of number of loci, and produced ambiguous results (Bayzid and Warnow 2013). Because we simulated a new species tree for each replicate, we are able to make more general observations regarding relative performance. As expected, the relative performance of *BEAST is higher when branch lengths are shorter. The relative performance of *BEAST is also higher as the number of loci is increased (Fig. 2).

The primary measure we chose to explore statistical accuracy, relative species tree error, incorporates both branch length and topological error. This measure is particularly relevant for molecular dating and downstream analyses of macroevolution and ecology. For example, the ${\text{PD}}_{\text{C}}$ measure of phylogenetic diversity and the BiSSE model of binary character influence on birth and death rates both assume accurate tree topologies and branch lengths (Maddison et al. 2007; Cadotte et al. 2008). When inferring species trees with shorter branch lengths, *BEAST using tens of loci outperformed supermatrix methods by this measure, even when other methods were able to utilize thousands of loci (Fig. 4a).

If instead branch lengths are irrelevant for a study, *BEAST still outperformed other methods for a given number of loci when inferring the topology of shallow species trees (Fig. 4e). However, when using thousands of loci, other methods were able to outperform *BEAST because *BEAST was restricted to tens of loci.

For certain species trees concatenation is statistically inconsistent (Roch and Steel 2015) and might not outperform *BEAST even when using thousands of loci. For deeper phylogenetic trees, *BEAST performed similarly to the Bayesian supermatrix method, which in turn was superior to RAxML given larger numbers of loci (Fig. 4b and f). Unpartitioned concatenation is known to potentially change the branch lengths and topology of estimated trees relative to partitioned concatenation (Kainer and Lanfear 2015), so this difference may be due to method configuration rather than a quality of the statistical method employed (maximum likelihood). Regardless, as *BEAST requires substantially more computational time, concatenation methods may be preferable in this case.

Multispecies coalescent methods assume free recombination between loci, and no recombination within loci. Short sequences dispersed throughout a genome, including RAD tags, can be justifiably used with coalescent methods as violations of both assumptions are likely to be limited. However, shortcut coalescence methods like MP-EST suffer from high gene tree estimation error when applied to these short sequences (Mirarab et al. 2014a; Springer and Gatesy 2016). In our study, MP-EST was inferior to *BEAST and similar to concatenation when inferring shallow phylogenies using short, RAD tag-like sequences (Fig. 4e). When inferring deep phylogenies MP-EST was inferior to both *BEAST and concatenation (Fig. 4f), despite the longer loci used for those simulations.

Newer fast multispecies coalescent methods such as ASTRAL (Mirarab et al. 2014b) and SVDquartets (Chifman and Kubatko 2014) may perform better at inferring species tree topology—the latest iteration of ASTRAL is both faster and less sensitive to gene tree error than MP-EST (Mirarab and Warnow 2015). However because these methods compute unrooted species trees without branch lengths, they cannot be compared with other methods using relative species tree error or rRF scores.

### Practical Implications for Applied Phylogenetics

Systematists can use the results of this study as a guide to choosing an appropriate phylogenetic method. If both *a priori* estimates or boundaries of root height (clade age) and extant effective population sizes are available for a particular study system, and the Yule process is a good fit for that system, an approximate estimate of branch length in coalescent units can be made before selecting a particular method.

Previous work has shown that the expected mean branch length of a Yule tree is equal to 1/2λ (Steel and Mooers 2010). Under the Yule model this value is related to the expected root height:

(5) $$\frac{1}{2\lambda }=\frac{R}{2(H_{n}-1)},$$

where R is the expected root height and $H_{n}$ is the $n^{\text{th}}$ harmonic number (where n is the number of species). The expected branch length $\bar{b}$ in coalescent units of $\tau {\left(2N_{e}\right)}^{-1}$ is therefore:

(6) $$\bar{b}=\frac{1}{2\lambda }\cdot \frac{1}{2N_{e}}=\frac{1}{4}\cdot \frac{R}{H_{n}-1}\cdot \frac{1}{N_{e}}.$$

The mean root height of the shallow simulations was 0.01315, and the mean of the reciprocal extant population sizes $1/N_{e}$ was 302.05. The approximate branch length in coalescent units based on these averages is:

(7) $$\bar{b}=\frac{1}{4}\cdot \frac{R}{H_{n}-1}\cdot \frac{1}{N_{e}}=\frac{1}{4}\cdot \frac{0.01315}{H_{n}-1}\cdot 302.05=0.578.$$

This approximate value is quite close to the sample mean of simulated branch lengths; $0.539\tau {\left(2N_{e}\right)}^{-1}$. Based on the results of experiment 2, this value of $\bar{b}$ is towards the lower bound of the crossover zone, and *BEAST will be preferred under most conditions (Fig. 2). As with experiment 1, parameters which were kept constant may move this crossover point to be more or less favorable to *BEAST.

The results of experiment 3 will inform researchers with access to phylogenomic data in the order of hundreds or thousands of loci on how to select an appropriate inference method. If branch lengths are at all important, either for reporting divergence times or for downstream analyses which require a species tree, using a subset of loci with *BEAST will be superior to using all loci with other methods tested for shallow phylogenies (Fig. 4a). If instead only the topology of the species tree is of interest, concatenation methods may be superior to fully Bayesian multispecies coalescent methods like *BEAST until improvements can be made to their computational performance (Fig. 4e and f).

### Open Questions in Phylogenomic Inference

Our results point to a number of areas for further research into the performance of species tree inference.

When using a single locus for species tree inference, experiment 2 shows Bayesian supermatrix analysis outperforming *BEAST for trees with longer branch lengths. This may be due to the population size priors used in *BEAST. However, our many-method comparison shows similar performance for both methods given species trees with long branch lengths. Because deep phylogenetic trees from experiment 3 were longer than the longest trees from experiment 2, this may point to a zone of intermediate branch lengths where *BEAST performs poorly given a single locus.

For all simulations we assumed a constant rate of speciation, however many lineages of life have undergone rapid radiations. It may be that when inferring species trees of clades containing ancient rapid radiations the performance of phylogenetic methods is closer to the shallow simulations than the deep simulations, and hence *BEAST becomes the preferred method.

Sequence alignments were generated and subsetted uniformly for all simulations regardless of the number of loci used for each analysis. In practice, researchers may reasonably choose longer, more informative loci when subsetting phylogenomic data sets for use with methods like *BEAST which are computationally intensive. This may improve the relative performance of *BEAST given a subset of the most informative loci relative to supermatrix or summary methods using thousands of loci.

However, whole proteins and transcripts can span genomic regions hundreds of thousands of nucleotides long, so recombination within loci will be common. The use of whole proteins or transcripts with coalescent methods has been dubbed “concatalescence” to reflect this violation (Gatesy and Springer 2013, 2014). If these long sequences are instead split into their constituent exons, the assumption of free recombination between loci may be violated due to short intronic distances. Further studies are needed to resolve which violation is less harmful to statistical accuracy.

### Conclusion and Future Directions

The multispecies coalescent is applicable to a wider range of conditions than has been suggested by more limited simulation studies. Our results confirm that the multispecies coalescent is especially suited to the estimation of shallower evolutionary relationships. We have also demonstrated that scaling of *BEAST to problems involving hundreds of loci is feasible, however very long chains and/or crude parallelization approaches need to be employed.

We anticipate that the increasing availability of phylogenomic sequence data will motivate further improvements to the computational efficiency of fully Bayesian inference under the multispecies coalescent model, which should allow for analysis of hundreds or even thousands of loci across tens or hundreds of species. These improvements will need to scale efficiently on many-core systems such as cluster supercomputers, as such systems offer vastly greater computing power than any desktop workstation.

## Supplementary Material

Supplementary material, including tables and figures can be found in the Dryad Data Repository: http://dx.doi.org/10.5061/dryad.02tf9.
